# Current Insights and Latest Updates in Sperm Motility and Associated Applications in Assisted Reproduction

**DOI:** 10.1007/s43032-020-00408-y

**Published:** 2020-12-07

**Authors:** Reyon Dcunha, Reda S. Hussein, Hanumappa Ananda, Sandhya Kumari, Satish Kumar Adiga, Nagarajan Kannan, Yulian Zhao, Guruprasad Kalthur

**Affiliations:** 1 Department of Clinical Embryology, Kasturba Medical College, Manipal, Manipal Academy of Higher Education, Manipal, 576104 India; 2 Division of Reproductive Endocrinology and Infertility, Department of Obstetrics and Gynecology, Mayo Clinic, 200 1st St SW, Rochester, MN 55905 USA; 3 Department of Obstetrics and Gynecology, Assiut University, Assiut City, Egypt; 4 Division of Experimental Pathology and Laboratory Medicine, Department of Laboratory Medicine and Pathology, Mayo Clinic, Rochester, MN 55905 USA; 5 Center for Regenerative Medicine, Mayo Clinic, Rochester, MN 55905 USA; 6 Mayo Clinic Cancer Center, Mayo Clinic, Rochester, MN 55905 USA; 7 Department of Laboratory Medicine and Pathology, Mayo Clinic, Rochester, MN 55905 USA

**Keywords:** Assisted reproductive technology, Male infertility, Sperm motility, Sperm motility enhancers

## Abstract

Spermatozoon is a motile cell with a special ability to travel through the woman’s reproductive tract and fertilize an oocyte. To reach and penetrate the oocyte, spermatozoa should possess progressive motility. Therefore, motility is an important parameter during both natural and assisted conception. The global trend of progressive reduction in the number and motility of healthy spermatozoa in the ejaculate is associated with increased risk of infertility. Therefore, developing approaches for maintaining or enhancing human sperm motility has been an important area of investigation. In this review we discuss the physiology of sperm, molecular pathways regulating sperm motility, risk factors affecting sperm motility, and the role of sperm motility in fertility outcomes. In addition, we discuss various pharmacological agents and biomolecules that can enhance sperm motility in vitro and in vivo conditions to improve assisted reproductive technology (ART) outcomes. This article opens dialogs to help toxicologists, clinicians, andrologists, and embryologists in understanding the mechanism of factors influencing sperm motility and various management strategies to improve treatment outcomes.

## Introduction

The human spermatozoon is an extremely specialized motile cell with a highly condensed nucleus and scanty cytoplasm. Even though transcriptionally and translationally inactive, it has precise metabolic pathways which are fundamental for fertilization to take place. After its production in the seminiferous tubules, the sperm undergoes maturation in the epididymis and then travels through the female reproductive tract to facilitate the transfer of paternal genome into the oocyte. Spermatozoa, which are deposited in the vagina during coitus, must reach the site of fertilization—namely ampullary site of the uterine tube (also known as fallopian tube). To reach the ampulla, in addition to the self-propelling properties of spermatozoa (forward progressive motility), the female reproductive tract assists in this process, which is regulated by the female reproductive hormones. Therefore, among all the semen parameters, sperm motility is considered to be a strong predictive marker of male fertility potential [1]. Based on studies with excised human uterus and tubes, it is estimated that human spermatozoa travel an average distance of approximately 19 cm and undergoes several physiological and biochemical changes before meeting an oocyte [2].

Based on the pattern of movement and velocity, spermatozoa can be graded as progressively motile, non-progressive (exhibiting only lateral head displacement), and immotile. As per the recent guidelines of the World Health Organization [3], the reference values for human semen characteristics are specified in Table 1. Men with ejaculates showing less than 40% total motile or 32% progressively motile spermatozoa are considered to be asthenozoospermic, a condition characterized by disorders in sperm motility [4]. Asthenozoospermia is considered as one of the predominant contributing factors for male infertility [5]. The purpose of this review is to recapitulate the physiology and signaling cascade of sperm motility, common disorders and factors which affect motility, importance of motility in assisted reproductive technology (ART), and possible approaches to improve motility in spermatozoa.

**Table 1:** Lower reference limits for semen characteristics [3]

Parameters	Lower reference limit
Volume (mL)	1.5
Total sperm number (10^6^ per ejaculate)	39
Sperm concentration (10^6^ per mL)	15
Total motility (Progressive + Non progressive, %)	40
Progressive motility (%)	32
Vitality (live spermatozoa, %)	58
Sperm morphology (normal forms, %)	4
pH	≥ 7.2

## Structure of Human Spermatozoa

**Fig. 1:**
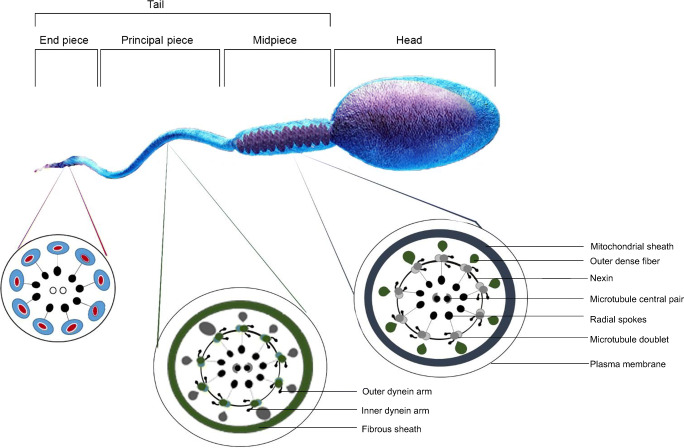
Structure of mature human spermatozoon and cross section of flagella at various segments of the tail

Sperm motility is controlled by its complex, structural and molecular signaling mechanisms. Broadly, spermatozoa are divided into three main parts—the head, which contains nuclear material; the tail or flagellum, which contains the machinery needed to propel the spermatozoa forward; and neck or connecting piece, which connects the head to flagellum (Fig. 1). The flagellum can be further segregated into the midpiece (containing cellular organelles like mitochondria), principal piece, and end piece [6].

The pivotal part of the sperm flagellum is the axoneme, which originates at the connecting piece and terminates at the end piece. It is composed of 9 microtubule doublets and a central pair, commonly termed the *9+2* arrangement (Fig. 1). These 9 microtubules are controlled by nexin links that connect to the central pair by radial spokes. Inner and outer axonemal dynein arms, which are key to acquiring motility in sperm, project from the microtubule doublets. The dynein arms help in sliding the microtubule doublets by consuming adenosine triphosphate (ATP) [7].

In mammalian sperm, the axoneme is covered by accessory structures, such as the outer dense fibers (ODFs), fibrous sheath (FS), and mitochondrial sheath (MS). In the midpiece, the axoneme is surrounded by ODFs and MS. In humans, the MS is spirally wound around the axoneme, which provides energy in the form of ATP required for sperm motility. In the principal piece, the axoneme is surrounded by ODFs and FS. The ODFs are petal-shaped structures that lie directly above the axoneme microtubule doublets which progressively decrease in diameter from base to tip of the principal piece. It is the principal piece that renders shape and flexibility to the tail. In addition to this, the principal piece provides room for signaling proteins that regulate motility and those involved in capacitation and hyperactivation. No accessory structures between the axoneme and plasma membrane are present in the end piece [8].

## Risk Factors Affecting Sperm Motility

**Fig. 2:**
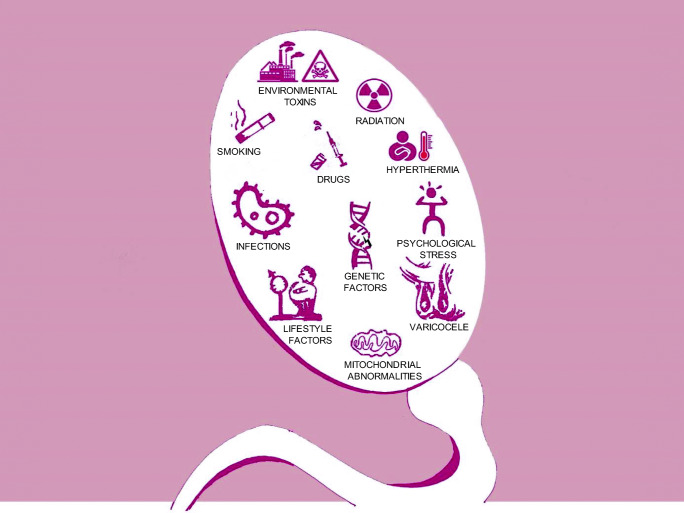
Factors which affect human sperm motility

Human ejaculate is highly heterogenous with respect to motility, morphology, and other functional characteristics of spermatozoa. Globally, about 20 to 30% of infertility cases are due to sperm-related problems in men of reproductive age [9]. There is strong evidence to suggest that lifestyle and other environmental factors contribute considerably to semen disorders leading to male infertility (Fig. 2). Even though these factors affect different semen parameters, in the context of this article, we focus mainly on the important factors that are known to affect sperm motility.

### Varicocele

Varicocele is a common chronic pathology in men, caused by abnormal dilatation of veins in the scrotum that leads to impairment of regular semen parameters. A systematic review and meta-analysis revealed that varicocele is strongly correlated with poor semen profile [10]. A compromised testicular microenvironment due to elevated levels of highly reactive oxidants and reduced levels of antioxidant is commonly observed in this condition [11]. Plenty of evidence in the literature suggests that varicocele is associated with poor sperm motility [10–12]. A high percentage of inactive mitochondria [13], abnormal expression of mitochondrial proteins [14], decrease in ATP levels [13], and altered calcium signaling cascade [15] in spermatozoa of men with varicocele has been reported in the literature. Significant decrease in kinematic parameters, such as curvilinear velocity (VCL), straight line velocity (STR), and amplitude of lateral head displacement (ALH), was observed in men with varicocele [16]. However, it remains inconclusive whether the surgical procedures like varicocelectomy improves the sperm motility [17–19]. Further, efforts to improve the semen characteristics using micronutrient and antioxidant supplements have shown some promising results [20, 21].

### Genetic Abnormalities Associated with Sperm Motility Disorders

Sperm motility depends upon the flagellar structure and function. Several reports indicate the association of poor motility with genetic defects [22–24]. The most common conditions are primary ciliary dyskinesia (PCD) and Kartagener syndrome. These are autosomal recessive disorders with an incidence of 1 in 20,000 and 1 in 30,000, respectively [24, 25]. In these conditions, spermatozoa lack motility due to defective dynein arms, with half of the cases having defects in the formation of the central pair complex and radial spokes. Lack of sperm motility is observed in 90% of PCD disease conditions and involves the outer and inner dynein arms or both of the PCD-associated genetic mutations of the dynein genes [23]. Dysplasia of FS is one of the structural flagellar abnormalities observed in spermatozoa, characterized by hyperplasia and hypertrophy of the FS. In this condition, the midpiece is invaded by the hypertrophied FS, and the annulus is predominantly not formed [22]. Dysplasia of FS was shown to have a familial predisposition in 20% of cases. However, to date, there is no consensus about the genetic background of dysplasia of FS [26].

### Mitochondrial DNA Mutations and Sperm Motility

Mitochondria are an important source of energy required for sperm motility. Usually, abnormalities of the MS or mitochondrial membrane integrity are associated with sperm motility disorders [27]. Deletions or mutations in mitochondrial DNA are correlated with elevated oxidative stress, sperm immotility, and male infertility [28, 29]. In addition, researchers have identified polymorphic mutations in genes encoding the oxidative phosphorylation complexes and transfer RNA of mitochondrial DNA associated with low sperm motility [30]. A missense mutation (C119941) in the mitochondrial *ND4* (NADH dehydrogenase 4) gene has also been reported as the reason for low sperm motility [31]. Further studies are necessary to unravel the genetic association between sperm motility using advanced techniques like whole exome sequencing or appropriate animal models.

### Antisperm Antibodies

It has been postulated that antisperm antibodies (ASAs), an autoimmune condition, can significantly affect male fertility due to poor sperm motility [32]. Roughly, 6 to 11% of male patients with infertility are known to have ASAs in their seminal plasma [33]. ASAs are known to hinder progressive motility and block sperm-egg interaction. This autoimmune condition can either be spontaneous or idiopathic and is mostly found in homosexual men and patients with varicocele, testicular trauma, mumps, orchitis, congenital absence of the vas, and spinal cord injury, as well as in those who have undergone vasectomy [34]. The mechanism by which ASAs cause reduced sperm motility is mainly due to the entangling of the spermatozoa at specific regions (head to head, head to midpiece, head to tail, or non-specific binding), due to the binding of immunoglobulins to sperm surfaces. Several methods to reduce ASA in the semen have been explored. Corticosteroid treatment [35], proteolytic enzyme treatment [36], use of immunobeads [37], and immunomagnetic sperm separation methods [38] have shown significant beneficial role. However, in assisted conception and assisted reproduction, the sperm-washing process may be sufficient to get rid of ASAs [39, 40].

### Sexual Abstinence

Human spermatozoa gain motility potential during their epididymal transit, which is around 2–11 days [41]. During their transport and storage in epididymis, spermatozoa undergo series of physiological and biochemical changes to acquire fertilizing ability. Considering these facts, WHO has recommended 2–7 days as an ideal abstinence time for assessing the semen parameters [3]. However, it is important to note that prolonged sexual abstinence can lead to accumulation of spermatozoa in epididymis, which has limited ability to provide a conducive environment to spermatozoa for long time [42]. Elevated oxidative stress and poor antioxidant defense in the epididymal microenvironment may compromise the sperm parameters under such circumstances. Studies suggest that spermatozoa are highly vulnerable to oxidative stress due to the elevated level of polyunsaturated fatty acids (PUFA) in the spermatozoa membrane [43, 44]. A study undertaken by Comar et al. [42] in 2458 men reported a significant negative effect of abstinence on sperm viability, motility, and mitochondrial membrane potential. Considering the poor sperm quality with prolonged abstinence, discrepancies between researchers over the ideal abstinence for therapeutic insemination procedures continue. Shen et al. [45] reported that ejaculates collected from men with short abstinence (1–3 h) period compared to 3–7 days of abstinence showed increased sperm concentration and higher percentage of motile spermatozoa. Better sperm velocity, progressiveness, and hyperactivation were observed when the abstinence period was 2 h compared to 4–7 days [46]. This was true for oligozoospermic men as well. Dupesh et al. [47] reported that < 24 h abstinence in oligozoopermic men had highest percentage of progressively motile sperm and normal morphology. However, these studies reported lower sperm count and volume. Contrary to this, Elzanaty et al. [48] reported higher percentage of motile spermatozoa at 4–5 days as compared to 2–3 and 6–7 days of abstinence.

### Lifestyle and Demographic Factors Related to Sperm Motility

Environmental and lifestyle factors have shown to affect semen quality. Most of the studies have indicated strong correlation between alcohol intake and decreased sperm motility [49–51], whereas few studies have shown no effect [52, 53]. Studies have shown that abstinence from alcohol consumption can reverse the adverse effect of alcohol on motility [54, 55]. Tobacco inhalation is another common lifestyle factor known to contribute to compromised semen parameters. Inhalation of large number of toxins from tobacco smoking can affect spermatogenesis and semen quality, including motility. Even moderate smoking was shown to have significant adverse effects on progressive motility [56]. Tobacco smoke contains nicotine as the main hazardous chemical along with traces of tar, carbon monoxide, polycyclic aromatic hydrocarbons, and heavy metals [57]. However, an in vitro experiment demonstrated that nicotine and cotinine are not responsible for the decrease in motility. Hence, other components, such as carbon monoxide, hydrogen cyanide, alcohols, ammonia, volatile hydrocarbons, aldehydes, and ketones may result in decreased sperm motility [58]. The decrease in motility could also be due to the epididymal dysfunction in smokers or elevated oxidative stress in the testicular environment [57]. High malondialdehyde (MDA) and protein carbonyl levels and low levels of glutathione S-transferase (GST) and reduced glutathione (GSH) were reported in seminal plasma and spermatozoa of smokers [59]. Other factors such as high body mass index [60], meat intake frequency [61], intense physical activity [62], prolonged cell phone [63] and laptop usage [64], and lack of sleep [65] are considered as potential risk factors for decrease in sperm motility. Therefore, lifestyle modification such as consuming nutritious diet, regular exercise, and withdrawal from substance abuse, smoking, and alcohol consumption can improve semen parameters considerably [54, 66].

### Drugs Affecting Sperm Motility

There is sufficient evidence in the literature indicating the deleterious effect of chemotherapeutic drugs on spermatogenesis in cancer patients [67–69]. While the negative effect of chemotherapeutic drugs on sperm production is well documented, their effect on sperm motility remains unclear. Animal studies have demonstrated that anticancer drugs such as vincristine, cisplatin, and cyclophosphamide impair epididymal function, thereby affecting sperm motility [70]. Literature indicates that other commonly used medications have considerable adverse effects on sperm motility. In vitro studies have shown that psychotropic drugs (imipramine hydrochloride, desmethylimipramine, chlorpromazine, trifluoperazine, and nortriptyline hydrochloride) act as potent inhibitors of sperm motility [71]. Antiepileptic drugs (phenytoin, carbamazepine, and valproate) had adverse effects on motility both in vivo and in vitro [72]. Consumption of high amounts of acetaminophen (commonly known as paracetamol), an antipyretic, has also shown to decrease sperm motility [73]. Lansoprazole, a proton pump inhibitor used to treat gastric illness, has shown to reduce the motility due to its calcium quenching effect or decreased Na^+^-K^+^-ATPase activity [74]. Moderate consumption of aspirin, a non-steroidal anti-inflammatory drug (NSAID), is known to demonstrate similar effects in young men [75]. In addition, regular consumption of recreational drugs, such as marijuana, is shown to affect spermatogenesis as well as sperm motility [76]. However, there are no clear reports in the literature to suggest whether the effects of these drugs on motility are reversible or irreversible.

### Radiation

Radioactivity (natural or by human activity) is an inevitable element surrounding humans. Exposure to radioactivity may be primarily due to occupational environments (mine fields, medical setups, flights at altitude of above 10,000 m) or patients who receive radiation as a part of diagnostic or therapeutic procedure. Some geographic locations may naturally have high radioactivity in their surroundings in the form of gases, such as radon or radionuclides in rocks [77]. Testes are considered extremely sensitive to radiation-induced damage. Earlier studies have shown that exposure to radiation can drastically affect motility and morphology and cause intense vacuolization in human spermatozoa [77, 78]. Studies conducted in people exposed to radiations from the atomic bombings of Hiroshima and Nagasaki [79] and the Chernobyl incident [80] have revealed poor motility in the spermatozoa of ejaculates from these men. Even though the mechanism behind the radiation-induced defective sperm motility is not clearly elucidated yet, significant reduction in the expression of cation channel of sperm associated1 (*CatSper1)* and cation channel of sperm associated2 (*CatSper2)* genes [81], and other sperm motility–associated proteins were observed in mice [82]. Kesari et al. [83] reported that as low as 850 MHz of non-ionizing radiation impaired sperm motility in human. The decrease in motility and its recovery from radiation-induced assault is dose-dependent. Exposure to a threshold of 0.1 Gy of ionizing radiation caused significant decrease in sperm parameters, which was reversible after 9–18 months. However, exposure to > 3 Gy caused permanent infertility [84]. Considering the extreme sensitivity of the testicular tissue to radiation-induced damages, it is a common practice to use lead shields to minimize the exposure to testes during radiotherapy.

### Heat Exposure

Scrotal temperature is 2–5 °C lower than the core body temperature in mammals, which is essential for normal spermatogenesis to take place. It is suggested that high heat exposure may perturb regulation of intrascrotal temperature and increase intratesticular temperature, both of which have drastic effects on semen quality [85]. A study performed on mice demonstrated that heat exposure deleteriously affected sperm motility and morphology and resulted in delayed conception [86]. Gong et al. [87] demonstrated that heat stress decreases sperm motility by downregulating mitochondrial activity and decreasing ATP levels. Transient scrotal hyperthermia was shown to cause reversible reduction in proteins required for spermatogenesis, gamete interaction, and motility [88]. Decreased antioxidant level, mitochondrial degeneration, and alteration in protein expression pattern have shown to be associated with poor motility [89]. Heat stress is also known to cause dephosphorylation of glycogen synthase kinase-3α (GSK), a negative regulator of sperm motility and interference in mitochondrial remodeling. Therefore, men exposed to higher temperatures due to their occupation (bakers, foundry workers, welders) [90] and other factors which increase the intratesticular temperature such as sedentary work habits [91], wearing tight under garments [92], and frequent sauna use [93] may have an increased risk of defective sperm motility.

### Environmental Factors and Sperm Motility

Due to a rapid increase in industrialization and urbanization, our environment is highly polluted by various natural and synthetic chemical agents generated by industrial or agricultural activities. Environmental contaminants, especially those with endocrine-disrupting function, are suspected to interfere with normal spermatogenesis and decrease the semen quality and human fertility. The published data available in the literature show that various environmental chemicals, such as pesticides, polychlorinated biphenyls [94], bisphenol A [95], glycol ethers [96], perfluoronated compounds [97], dioxins and dioxin-like compounds [98], phthalates [94], heavy metals [99], dichloro-diphenyl-trichloroethane [100], and plasticizers [101] have adverse effects on sperm motility.

### Psychological Stress

Psychological stress is an “emotional experience” accompanied by several biochemical, physiological, and behavioral changes or responses. During the events of stress, corticosterone elevation suppresses testosterone and inhibin levels, thereby causing alteration in testicular microenvironment [102]. Studies have shown a negative effect of psychological stress with sperm progressive motility [103]. Stress can affect male fertility through different mechanisms, mostly through altering testosterone secretion and through disruption of the blood-testis barrier [104]. Inhibition of the hypothalamic-pituitary-gonadal axis via the inhibitory effect of gonadotropin-inhibitory hormone [105] and activation of the hypothalamic-pituitary-adrenal axis by producing an inhibitory effect on hypothalamic-pituitary-gonadal and Leydig cells, consequently impairs spermatogenesis [106]. The effect of psychological stress on reduced sperm motility could also be due to increased nitric oxide (NO) level. Excessive NO generated during psychological stress can produce peroxinitrite radicals (ONOO^−^) that causes oxidative damage and mitochondrial dysfunction, thereby causing reduced motility [107]. Nevertheless, it is encouraging to note that the impact of psychological stress on sperm motility or quality seems to be modifiable and reversible [104].

### Infections and Sperm Motility

Microbial infection is also known to affect reproductive outcome. Sexually transmitted diseases caused by bacterial, fungal, and viral pathogens can significantly decrease semen quality and can be a contributing factor for male infertility [108]. Presence of small amount of these pathogens is shown to decrease sperm motility. Experimental evidence suggest that bacteriospermia decreases sperm motility significantly due to bacterial infections, leucocyte accumulation (leukocytospermia), antibody buildup, inflammation and oxidative stress [109]. *Chlamydia trachomatis* [110] and *Ureaplasma* sp. [111] infections are reported to affect sperm motility. Similarly, Burrello et al. [112] reported that infections caused by *Candida albicans*, a pathogenic yeast, decreased sperm motility significantly by reducing mitochondrial membrane potential and increasing apoptosis of human spermatozoa in vitro. Pathogens like hepatitis B virus [113], human papillomaviruses [114], herpes simplex viruses [115], and adeno-associated virus [116] were associated with significant reduction in sperm parameters, especially progressive motility. A recent report suggests that infection with SARS-Cov-2 coronavirus in men can lead to low sperm count and poor motility for 90 days following infection [117]. It is not explicit if sperm motility improves following any specific (antibacterial/antiviral/antifungal) therapy in men with these infections. However, Garolla et al. [118] observed improvement in progressive motility in HPV-infected men after HPV adjuvant vaccination.

## Signaling Mechanisms Involved in Sperm Motility

**Fig. 3:**
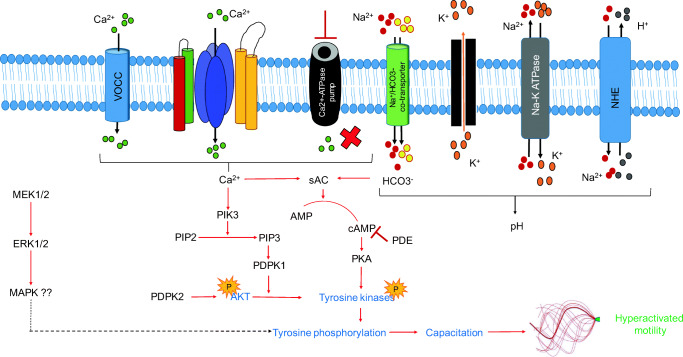
Signaling mechanisms involved in regulation of motility in human spermatozoa. AKT, alpha serine/threonine-protein kinase; AMP, adenosine monophosphate; ATP, adenosine triphosphate; cAMP, cyclic adenosine monophosphate; Ca^2+^, calcium ion; ERK1/2, extracellular signal regulated kinase; H^+^, hydrogen ion; HCO_3_^−^, bicarbonate ion; K^+^, potassium ion; MAPK, mitogen activated protein kinase; MEK1/2, mitogen activated protein kinase kinase ; Na^2+^, sodium ion; NHE, sodium-hydrogen exchanger; P, phosphorylation; PDE, phosphodiesterase; PDPK1, 3-phosphoinositide dependent protein kinase 1; PDPK2, 3-phosphoinositide dependent protein kinase 2; PIK3, phosphoinositide 3-kinase; PIP2, phosphatidylinositol 4-5-bisphosphate; PIP3, phosphatidylinositol (3,4,5)-triphosphate; PKA, protein kinase A; sAC, soluble adenylate cyclase; VOCC, voltage-dependent calcium channel

Motility is a complex physiological property of spermatozoa, which is dependent upon many extrinsic and intrinsic factors (Fig. 3). Several complex signaling pathways contribute to sperm motility such as cyclic adenosine monophosphate (cAMP)/protein kinase A and phosphoinositide 3-kinase signaling, which are mediated through calcium ion (Ca^2+^), bicarbonate ion (HCO_3_^−^), or both [8]. The less investigated DAG-MAPK (ERK1/2) [Diacylglycerol-mitogen activated protein kinase (extracellular signal regulated kinase 1/ 2)] pathway is also involved in sperm motility signaling. This pathway is regulated at the membrane level by ion channels, such as CatSper and voltage-dependent calcium channel, and inhibited by Ca^2+^-ATPase, which promotes the Ca^2+^ influx process. Moreover, HCO_3_^−^, through the sodium (Na^+^)-bicarbonate (Na^+^-HCO_3_^−^) co-transporters, enhances the activation of downstream soluble adenylate cyclase (sAC) along with calcium, which promotes motility through elevation of cAMP (Fig. 3). Intracellular sperm pH regulation is also governed by hydrogen ion (H^+^) efflux and other ions, thereby activating the opening of CatSper and increasing the intracellular Ca^2+^ reservoir [119]. Hence, sperm motility is interconnected and associated with different physiological changes that are solely dependent upon the intracellular signaling pathways and post-translational modifications.

### Ca^2+^ as a First Messenger in Achieving Sperm Motility

Ca^2+^ is a fundamental messenger, which regulates capacitation, acrosome reaction, and hyperactivated motility. In human sperm, calcium influx is modulated by various mechanisms, such as increase in membrane permeability by loss of cholesterol from the sperm membrane, depolarization, inhibition of Ca^2+^-ATPase pump, activation of voltage-dependent calcium channels, and CatSper [8]. Compared to 100 to 200 nM resting calcium concentration that is needed for normal motility, an increase in the intracellular calcium level is needed for the spermatozoa to attain hyperactivated motility in the female reproductive tract [120]. The primary role of calcium is to activate sAC, which in turn further activates downstream signaling molecules. Inhibition of calcium influx by blocking Ca^2+^ channels has been demonstrated to cause male subfertility by preventing acrosomal exocytosis in humans [121].

### Role of cAMP

Cellular level of cAMP, a second messenger, is controlled by adenylate cyclases, which catalyze the conversion of ATP to cAMP with the release of inorganic phosphate [122]. Reduced level of cAMP is associated with reduced motility and infertility. G-protein-activated transmembrane adenylate cyclase and sAC are the two types of mammalian adenylate cyclases. Even though both types of adenyl cyclases are present in spermatozoa, motility appears to be solely regulated by sAC. sAC is activated by Ca^2+^ and bicarbonate directly and acts as a sensor for ATP, Ca^2+^, and HCO_3_^−^/carbon dioxide/pH at different intracellular sites. Importantly, sAC undertakes the task of converting ATP to cAMP, a secondary messenger that activates the protein kinase A pathway.

### Role of HCO_3_^−^ in Regulation of Sperm Motility

During the journey of sperm in the female reproductive tract, it is the bicarbonate ion that creates an alkaline environment for spermatozoa to achieve hyperactivated motility. Bicarbonate is transported into sperm through the sodium-bicarbonate cotransporters which is essential for capacitation and also a direct activator of sAC [123]. Upon its entry into the cell, it increases the intracellular pH and causes hyperpolarization of the membrane. Apart from the voltage-gated proton channel and Na^+^/H^+^ exchanger, transport of bicarbonate into the sperm contributes significantly to the regulation of pH [119]. Hence, Ca^2+^ and HCO_3_^***−***^ concentrations act through the sAC/cAMP/protein kinase A pathway to achieve hyperactivated motility. Levels of bicarbonate lower than the physiologic level in the ejaculate have also shown to cause reduction in sperm motility [124]. Sperm functional changes, such as capacitation and acrosome reaction, are imperative for successful fertilization, which is also regulated by HCO_3_^***−***^. As early as 1 min after bicarbonate exposure to spermatozoa, a peak in cAMP level can be observed, which rapidly evokes frequent flagellar beats and decreases beat asymmetry [125].

### Protein Tyrosine Phosphorylation

An increase in protein tyrosine phosphorylation is a hallmark of capacitation and hyperactivated motility in human spermatozoa. Most pathways studied in sperm motility belong to a family of protein tyrosine’s that get inevitably phosphorylated during the event of hyperactivation. Phosphorylation of both serine/threonine and tyrosine proteins in human spermatozoa has been reported during capacitation [126]. Among these, the tyrosine kinases Src fibrous growth factor receptor 1 (FGFR) and Abelson murine leukemia (ABL1) are known to be well associated with tyrosine phosphorylation in mammalian sperm. A-kinase-anchoring proteins (AKAP4), calcium binding tyrosine phosphorylation regulated proteins (CABYR), heat shock protein 90 (HSP90), and 95 kDa FS proteins that are present in the sperm flagellum have been defined as targets of tyrosine kinases [8].

## Sperm Motility and Infertility

Human ejaculate is highly heterogenous with respect to the types of cells present, motility pattern, and the quality of spermatozoa. Presence of immotile sperm in the ejaculate is not unprecedented and can arise because of testicular and/or epididymal dysfunction due to various risk factors, as discussed earlier. Clinically, presence of motile and morphologically normal sperm provides evidence for fertility potential among infertile patients. Based on the results from a study conducted on 4500 normozoospermic men from 14 different countries, the baseline for normal sperm characteristics was established by World Health Organization [127]. Like oligozoospermia [128], asthenozoospermia [29, 126] is also strongly correlated with infertility which suggests that motility is an equally important semen parameter to achieve pregnancy.

## Importance of Motility in Planning Therapeutic Insemination Procedures

To decide upon effective treatment for correcting infertility, infertility specialists depend upon semen parameters of male partner. Motility is one such parameter which plays an important role in deciding the appropriate therapeutic insemination option for the infertile couple. In general, to recommend intrauterine insemination (IUI), one should be able to extract at least 5 million motile sperm from the ejaculate; in vitro fertilization (IVF) is recommended when 2 to 5 million motile sperm can be extracted; and intracytoplasmic sperm injection (ICSI) is recommended when samples yield less than 2 million motile spermatozoa. Kinematic parameters, such as straight line velocity (VSL) and curvilinear velocity (VCL), have prognostic value in predicting the fertilization potential of spermatozoa [129, 130]. If the spermatozoa have a VCL greater than 65 μm/s and straight line velocity (STR) greater than 40 μm/s, IVF should be considered. If the velocities are lower than these values, to improve the fertilization rate ICSI is recommended, even if there is adequate percentage of motile spermatozoa to perform IVF [131].

### Motility and IUI Pregnancy

Sperm motility as a predictor of pregnancy in patients undergoing IUI has been a topic of discussion. Several studies have confirmed that the total progressively motile sperm count in fresh ejaculate does not have any prognostic value in predicting pregnancy outcome in IUI cycles [132–134]. However, the number of inseminated progressively motile spermatozoa (NIPMS) was considered a better predictive marker [135]. To achieve the best pregnancy rate in IUI, at least 5 million motile spermatozoa are thought to be essential [136]. A systematic review conducted by Ombelet et al. [137] proposed that IUI can still be tried with an NIPMS of more than 1 million before directing the patient to IVF. However, the pregnancy rate in such circumstances is expected to be low. In a retrospective study comprised of 1166 couples undergoing IUI cycles, Lemmens et al. [138] found that pregnancy probability significantly decreased when the NIPMS was less than 1 million. In the case of insemination with cryopreserved semen samples, total number of motile sperm less than 20 million significantly decreases pregnancy rate [139], possibly due to the poor functional competence of frozen-thawed spermatozoa.

### Motility and IVF Pregnancy

It has been established that spermatozoa having at least 30% motility and 15% progressive motility are required to perform IVF [140]. Sperm motility is known to have a strong correlation with IVF success and pregnancy outcome [141]. Superior sperm kinematic parameters are also considered to improve IVF outcome. The percentage of motile spermatozoa with an average path velocity (VAP) between 10 and 20 m/s were known to significantly increase success rates during IVF [142]. Donnelly et al. [141] reported that values for VAP, VSL, and VCL were significantly higher in samples that produced > 50% fertilization, indicating positive correlation between progressive motility and fertilization outcome. Contrary to these reports, Moghadam et al. [143] reported that motility did not enhance fertilization rate or improve pregnancy outcome through IVF. Further, with the advent of ICSI, the use of conventional IVF practice has drastically reduced [144].

### Motility and ICSI

Motility is an important parameter in ICSI, as it helps the embryologist in picking a viable spermatozoon for microinjection, especially in case of absolute asthenozoospermia or if the spermatozoa are retrieved by testicular sperm aspiration. Apart from poor fertilization due to injection of non-viable spermatozoa into the oocyte, lack of motility in the sample may have an indirect negative effect on the fertilization outcome due to the delay in completion of microinjection procedure. Identifying a suitable viable spermatozoon is challenging which may potentially cause delay in completion of the ICSI procedure. Bartolacci et al. [145] in a recent retrospective study of 1266 ICSI cycles reported that low sperm motility and concentration compromise fertilization and blastocyst rates but have no impact on the implantation potential of the obtained blastocysts or rate of top quality blastocyst formation. These results are consistent with a study conducted by Mazzilli et al. [146] that included 1219 couples undergoing ICSI cycles with preimplantation aneuploidy tests. It was proposed that poor sperm motility could lower fertilization rates and impair the developmental competence of early embryos but had no effect on pregnancy rate or euploidy of the obtained blastocysts, whereas Miller and Smith [147] reported that defective motility is not linked to poor fertilizing ability in ICSI. It is instead related to developmental arrest at the cleavage stage (day 3 embryos) or decreased rate of blastocyst formation. Sperm motility was also shown to be positively associated with the quality of the sperm nucleus [148], thus showing an added benefit to selecting the most motile sperm.

### Improvement in Sperm Motility In Vivo

Efforts to ameliorate testicular sperm output or semen quality have been explored in the past with various approaches, however, with minimum success. Oral supplementation of synthetic drugs, vitamins, trace elements, and other natural compounds have been used historically for enhancing sperm motility in men (Table 2). Among these, the most widely used approaches are based on mitigating the oxidative stress in the testicular microenvironment using antioxidants. Few studies have shown the beneficial effects of oral supplementation of antioxidants or trace elements in boosting sperm motility in infertile men. Antioxidants such as vitamin E [149], coenzyme Q_10_ [150], l-carnitine [151], vitamin C [152], and lycopene [153], alone or in combination with trace elements like selenium [154] or zinc [155], have demonstrated improvement in sperm motility after oral administration. However, there are contradictory reports as well [156, 157]. In a recent article, Tsounapi et al. [158] reported significant improvement in sperm motility by using avanafil or combination of avanafil plus Profetil (mixture of micronutrients- l-carnitine, l-arginine, coenzyme Q_10_, vitamin E, zinc, folic acid, glutathione, and selenium). Pharmacological agents such as pentoxifylline [159] and avanafil [158], which are inhibitors of phosphodiesterase (PDE), and clomiphene citrate [160], an antiestrogenic molecule that increases endogenous serum follicle-stimulating hormone (FSH), luteinizing hormone (LH), and testosterone, are proven to enhance sperm motility in vivo.

**Table 2:** Drugs, bioactive compounds, and natural products used in empirical treatments to enhance human sperm motility

Agents		Mode of action
Drugs	Avanafil	PDE-5 inhibitor [158]
	Pentoxifylline	PDE inhibitor, increased cAMP, decreased ROS [185, 186, 159]
	Clomiphene citrate	Binding to estrogen receptor in hypothalamus; increased follicle-stimulating hormone and luteinizing hormone levels [187, 188]
Drugs along with bioactive compounds	Clomiphene citrate + vitamin E	Not known [189, 190]
	Pentoxifylline + zinc + folic acid	PDE inhibitor and antioxidant [191]
	Pentoxifylline + l-carnitine	PDE inhibitor and decreased ROS [192, 187]
Bioactive compounds alone or in combination	Vitamin C	Decreased ROS [193]
	Zinc	Increased metallothioneins and decreased oxidative stress [194, 155]
	Selenium	Not known [156]
	Coenzyme Q_10_	Decreased ROS [150, 195]
	l-Carnitine	Increased *GPX4* expression [196]
	Zinc + folate	Not known [197]
	Selenium + vitamin E	Increased *GPX4* expression, decreased oxidative stress [154, 198]
	Selenium N-Acetyl-cysteine	Not known [199]
	Fertilovit	Decreased ROS [200]
Herbal extracts	*Withania somnifera*	Enhanced enzymatic activity in seminal plasma, decreased oxidative stress [161, 201]
	*Tribulus terrestris*	Not known [202]
	*Mucuna pruriens*	Activated antioxidant defense system and physiologic stress [203]
	*Lepidium meyenii* (maca)	Not known [204]
	Speman (multiherbal formulation)	Not known [205]

*cAMP*, cyclic adenosine monophosphate; *PDE*, phosphodiesterase; *ROS*, reactive oxygen species

Natural compounds and crude plant extracts (individually or as multiherbal formulations) have also been tried extensively with impressive improvement in motility. In a triple blinded randomized clinical trial conducted on 100 idiopathic infertile men, Azgomi et al. [161] reported that extracts from *Withania somnifera* root improved sperm motility by 57%, similar to that of pentoxifylline. Even though there are not many studies on motility enhancement in human with plant extracts, several animal studies suggest the potential use of extracts in improving sperm motility. Nayak et al. [162, 163] have shown that ethanolic extract of *Moringa oleifera* leaves improves sperm motility in mice treated with cyclophosphamide. Similarly, other plant extracts like *Ruta chalepensis*, *Croton zambesicus*, *Shengjing* (a Chinese formula of plant extracts), *Panax ginseng*, *Nigella sativa* oil, *Phoenix dactylifera*, *Punica granatum* juice, *Asparagus recemosus*, *Tribulus terrestris*, *Mucuna pruriens*, and *Lepidium meyenii* are reported to increase sperm motility in animal models and humans [164]. Agrawal et al. [165] reported the use of Speman (The Himalaya Drug Company), a multiherbal formulation, which increased sperm motility in men with oiligozoospermia.

### Improvement in Sperm Motility In Vitro

Unlike other semen parameters, sperm motility is accessible to modulation under in vitro conditions, which serves as an advantage, especially for ART. A wide variety of compounds have been screened for motility enhancement in vitro (Table 3), among which the most popular agents are PDE inhibitors. Compounds like 8-methoxy isobutyl methyl xanthine (8-MeO-IBMX), rolipram, RS-25344, sildenafil, tadalafil, dipyridamole, isobutyl methyl xanthine (IBMX), ibudilast, tofisopam, etazolate hydrochloride, and papaverine were shown to increase sperm motility [166, 167]. Among all PDE inhibitors tested so far, caffeine and pentoxifylline are the two nonspecific PDE inhibitors that have been used most frequently as motility stimulants for human spermatozoa [166]. But, since caffeine and pentoxifylline are known to induce premature acrosome reaction, their clinical use as sperm motility enhancers has been limited [168]. Tardif et al. [167] screened 43 commercially available compounds with reported PDE inhibitor activity, among which 6 compounds (dipyridamole, ibudilast, tofisopam, etazolate hydrochloride, papaverine, and 8-MeO-IBMX) were able to significantly increase the percentage of total and progressive motility in human spermatozoa.

**Table 3:** Various pharmacologic and physiologic agents used for human sperm motility enhancement in vitro

Enhancers			Examples
PDE inhibitor	Selective inhibitors	PDE 1	8-MeIBMX [206]
		PDE 3	Trequinsin hydrochloride [207]
		PDE 4	Rolipram, RS-25344, tofisopam, etazolate hydrochloride [206, 167]
		PDE 5	Sildenafil, tadalafil[208–210]
		PDE10	Papaverine [211]
	Nonselective inhibitors		Dipyridamole [167]
			IBMX [212]
			Ibudilast [167]
			Caffeine [212]
			Pentoxifylline [213]
			Theophylline [212, 214]
Adenylyl cyclase enzyme stimulators			Adenosine, 2-deoxyadenosine [170, 215, 216]
			Forskolin, cAMP [217]
Calcium channel modulators (calcium chelators)			Diltiazem, flunarizine, verapamil [218]
Vitamins and antioxidants			Biotin [219, 220]
			Myoinositol [221]
			α-Tocopherol [222]
			Epigallocatechin gallate [223]
Peptides			Spermaurin [224]
Herbal medicines			*Tribulus terrestris* [183]
			*Mondia whitei* [184]
Co-culturing			Cumulus cells [178]
			Fallopian tubal cells [225]
			Vero cells [226]
Hormone and growth factors			Insulin and leptin [177]
			Platelet activating factor [227, 176]
			Follicular fluid [228, 229]
			Progesterone [230]
			Leukemia inhibiting factor [231]
			Thyroxine [173]
			Relaxin [175]
			Müllerian inhibiting substance [232]
			Human chorionic gonadotropin [174]
			Bradykinin [233]
High-energy molecules and prostaglandins			Creatine phosphate [234]

*cAMP*, cyclic adenosine monophosphate; *8-MeIBMX*, 8-methoxymethyl-3-isobutyl-1-methylxanthine; *IBMX*, 3-isobutyl-1-methylxanthine; *PDE*, phosphodiesterase

Apart from PDE inhibitors, treatment of human sperm with cAMP analogues, such as dibutyryl cAMP [169], adenosine, 2-deoxyadenosine [170], or activator of adenylate cyclase enzyme, such as forskolin [171], have shown a significant increase in total motility for a short duration. However, no significant difference was observed when spermatozoa were incubated over longer periods in vitro with dibutyryl cAMP or forskolin. Aitken et al. [171] reported that exposure of cryopreserved human spermatozoa to 2-deoxyadenosine resulted in significant increases in percentage of motility. However, there is limited information available in the literature on the potential application of these compounds in ART setup. Considering the role of protein kinases in the sperm motility pathway, LY294002, an inhibitor of phosphoinositide 3-kinase, was screened for its motility enhancement property. Several studies have shown potential stimulating effect of this compound on motility in humans [172]. However, a contradictory report showed notable differences in their potency [168].

Various physiologic agents, such as progesterone, thyroxin, and Müllerian inhibiting substance, were also tried and shown to improve sperm motility in vitro [173]. However, incubation of spermatozoa with Müllerian inhibiting substance led to inhibition of protein tyrosine phosphorylation, capacitation, and acrosome membrane exocytosis. Similarly, Moosavi et al. [174] reported an increase in sperm motility after incubation of rat spermatozoa with human chorionic gonadotropin, but the study lacks detailed investigation to understand the mechanism of action.

Growth factors, such as relaxin, platelet activating factor, leukemia inhibiting factor, and follicular fluid conditioned media, were also tried in vitro for sperm motility enhancement. Most of these agents improved sperm survival in vitro [175–177]. Co-culture of spermatozoa with cumulus cells under in vitro conditions increased sperm motility and longevity [178, 179]. However, the effect of these physiologic agents on sperm motility is dependent on sample type, concentration, and incubation duration [176]. At physiological concentrations, bradykinin, angiotensin I, II, and III, and acetylcarnitine exhibited a direct stimulating effect on sperm motility in vitro [180].

Various vitamins and antioxidants were shown to improve sperm motility and longevity by reducing in vitro oxidative stress [181]. Few herbal medicines, rich in antioxidants such as *Tribulus terrestris* extract [182, 183] and *Mondia whitei* [184], have been reported to enhance sperm motility in vitro. However, the major drawback is identifying the active principle from the crude extract and avoiding batch to batch variation in the plant products since the active constituents can vary with season and geographic location.

## Conclusion

Last several decades have seen a steady decline in sperm output and their functional properties such as motility in human mainly due to change in environmental and lifestyle factors. Therefore, adapting to a healthy lifestyle pattern may help in minimizing the loss of fecundity in men. Motility is a major determining factor for the successful pregnancy outcome, emphasizing the importance of research in the field of motility enhancement. Efforts to improve the sperm motility in ejaculated spermatozoa by empirical treatments with hormones, antioxidant supplements, and natural products have not shown consistent results. Considering the advantage of ex vivo manipulation of motility using pharmacological agents, specifically phosphodiesterase inhibitors, further extensive research in this aspect may prove beneficial to medically assisted or artificial insemination procedures. High-throughput screening approaches can accelerate identification of novel sperm motility enhancing agents. Further, it is essential to confirm that these motility enhancers do not exert any adverse effects on the developing embryo.

## Data Availability

Not applicable
